# A Novel Process for the Recovery of Betalains from Unsold Red Beets by Low-Temperature Enzyme-Assisted Extraction

**DOI:** 10.3390/foods10020236

**Published:** 2021-01-24

**Authors:** Claudio Lombardelli, Ilaria Benucci, Caterina Mazzocchi, Marco Esti

**Affiliations:** Department of Agriculture and Forestry Science (DAFNE), Tuscia University, via S. Camillo de Lellis snc, 01100 Viterbo, Italy; claudiolombardelli@libero.it (C.L.); catemaz@live.it (C.M.); esti@unitus.it (M.E.)

**Keywords:** red beet, betalains, tailored enzymatic mix, extraction yield, color attributes

## Abstract

Food waste management plays a central role in the circular economy. To our knowledge, only a few studies have investigated the use of unsold fruit and vegetables from supermarkets as a substitute source for the extraction of natural colorants. Thus, the aim of this paper was to suggest a green, tailored protocol that avoids the use of organic solvents for the recovery of betalains from unsold red beets for use as a food colorant. The recovery of such pigments was carried out by a tailored enzymatic mix, blended considering the polysaccharide composition of the beetroot cell wall; thus, it consisted of: cellulase (37%), xylanase (35%), and pectinase (28%). The enzyme-assisted extraction protocol was optimized, and the most suitable conditions (in terms of pigment yield and color attributes) for the recovery of betalains from unsold beets appeared to be: 25 U/g total dose of enzymatic mix, temperature 25 °C, and processing time 240 min.

## 1. Introduction

Color is a distinctive characteristic of food and beverages. It is considered a quality indicator that affects their appeal to consumers [1]. During processing and storage, many foods are prone to color loss, thus requiring the addition of colorants to restore it [2]. In the last decade, the demand for natural pigments (believed to be less harmful than synthetic colorants) is increasing worldwide [3]. Moreover, coloring foodstuffs are considered ingredients, and can be indicated as “coloring fruit- and plant extracts” on food labels. Other additives must be declared by an E-number [3]. In this context, the global market for natural food coloring (USD 3.71 billion in 2017) is expected to grow at a Compound Annual Growth Rate (CAGR) of 5.7% over the period of 2017–2023 [4].

From a Circular Economy approach, food waste (including unsold vegetables) is considered a promising source of natural colorants, sweeteners, antioxidants, and preservatives [5]. Among the natural pigments suitable as food coloring agents, the most attractive are anthocyanins, betalains, carminic acid, carotenoids, and chlorophylls [6]. Betalains are water-soluble, nitrogen-containing vacuolar pigments, derived from betalamic acid [7]. The nature of the residue associated with betalamic acid determines the classification of the pigment as red-violet betacyanin (λ ~ 540 nm) or yellow-orange betaxanthin (λ ~ 480 nm). Among betacyanins, the best-known is betanin, which gives the distinctive red color to red beets (*Beta vulgaris* ssp.); whereas, among betaxanthins, the best-known are vulgaxanthin, present in yellow beets (*Betavulgaris* L.), and indicaxanthin, found in cactus pears (*Opuntia ficusindica*) [8,9].

Although betalains and anthocyanins perform similar functions, they have never been reported in the same plant, seeming to be mutually exclusive in the plant kingdom. Betalains can also be found in roots, fruit, and flowers [10]. The food colorant known as ‘beetroot red’, extracted from beetroots, is commercialized in the European Union and United States as a food colorant (E162) [11], and it has several applications in food, including desserts and confectioneries, dry mixes, and dairy and meat products [12]. Globally, revenue from the beetroot powder market was valued at more than $15 billion in 2016 and is expected to grow at a CAGR of 5% between 2017 and 2027 [13].

Betalains have been recovered by various methods, such as diffusion-extraction, solid–liquid extraction, reverse osmosis, and ultrafiltration [14]. Betalain-containing materials are generally soaked or ground. Pigments can be water-extracted; although, in most cases, the use of methanol or ethanol solutions (20–50%) is required to complete the process. In addition, slight acidification of the extraction medium enhances betacyanin stability [15]. As described in the literature, the degree of cell membrane permeabilization is a major factor in determining extraction efficiency [16]. Recently, the development of an enzyme-assisted extraction procedure, based on tailored blending activities, has allowed us to improve plant cell-wall hydrolysis, thus raising the recovery yield of natural food colorants [5] and reducing the use of both solvents and energy [17]. It has been implemented for the extraction of colorants from grapes, alfalfa, marigold, safflower, strawberry, and red cherries [5,18]. Moreover, Naderi et al. [19] developed a selective, high-recovery approach based on the enzyme-assisted extraction of betalains from *Hylocereus polyrhizus* by the application of pectinase. 

This research suggests a tailored protocol, based on targeted substrate-enzymatic extraction, for the recovery of betalains from unsold red beets. The procedure consists of applying a specific enzymatic mix that was designed on the basis of the composition of fully ripened red beet cell walls. Moreover, the optimal conditions (total dose of enzymatic mix, temperature, and processing time) for betalain recovery yield, as well as the colorimetric parameters of the extract, were identified.

## 2. Materials and Methods

### 2.1. Red Beet

Unsold beetroot (*Beta vulgaris* ssp.), devoid of visual blemishes and contaminations, was supplied by a local market (Unicoop Tirreno S.C., Viterbo, Lazio region, Italy). The raw material was washed, crushed in an HR 2068 blender (Philips S. P. A., Milan, Italy), and stored at 2–4 °C for 30 mins before use for betalains extraction.

### 2.2. Enzymes and Chemicals

Pectinase from *Aspergillus niger* containing both polygalacturonase (PG) and pectin-liasic (PL) activity (83 I.U./g, [5]), cellulase from *A. niger* (CL, 18 I.U./g, [5]), and xylanase from *Aspergillus oryzae* (XL, 3 I.U./g, [5]) were supplied by Merck Group (Milan, Italy). All other reagents used were purchased from Merck (Milan, Italy). The specific activity of the abovementioned enzyme preparations was estimated using a previously described method [5].

### 2.3. Red Beet Analysis

#### 2.3.1. Chemico-Physical Parameters

Beetroot samples were dried at 30 °C for 5 days before dry matter (DM) and moisture determination [20]. pH and titratable acidity (TA) were measured following the method described by Desseva et al. [21] and Porto et al. [22]. 

#### 2.3.2. Total Sugar Content

Total sugar content was analyzed by measuring absorbance at 620 nm, following the Anthrone method [5,23,24]. The total sugar content (%) was determined through a calibration curve (A_620nm_ = 0.0047 * glucose concentration; *R*^2^ = 0.99) using a stock solution of glucose (50–250 μg).

#### 2.3.3. Cell Wall Polysaccharide Composition

The amount of cellulose, hemicellulose (xylans), and pectin in the beetroot was examined following Lombardelli et al. [5] after acid chemical hydrolysis [20]. 

### 2.4. Enzyme-Assisted Extraction of Betalains

The whole red beet samples (200 g) were washed, drained, and mixed (Phillip HR 2068 blender), and the resulting purée was used for the betalain extraction. The conditions of solid–liquid extraction were as follows: (i) 1:15 solid–liquid ratio between raw material and acetate buffer containing the multi-component enzyme mix (total dosage: 10, 18, 25, 38, and 50 U/g); (ii) extraction temperature of 25 or 45 °C, at pH 5.5 ± 0.1; (iii) varied extraction duration up to five hours, with samples taken at various processing times. The mixture was then filtered and the extracts were analyzed. Trials were conducted in triplicates.

### 2.5. Determination of Betalain Content

The amount of betalain, in terms of betacyanins (Bt) and betaxanthins (Bx), was determined spectrophotometrically by visible spectra (λ = 340–700 nm). Bt and Bx concentrations were calculated as described by Wruss et al. [25]:

Bt (or Bx) content (mg/L) = A × DF × MW × 1000/ε × i(1)

for Bt A_536nm_ and ε = 60,000 (molar extinction coefficient in Lmol^−1^cm^−1^); for Bx A_485nm_ and ε = 48,000 (Lmol^−1^cm^−1^); DF = dilution factor; molecular weight (MW) = 550 g/mol (for Bt) or 339 g/mol (for Bx); i = path length (1 cm). Samples were diluted and measured in triplicate.

### 2.6. Color Measurements

Color measurements were performed using a CR-5 colorimeter (Konica Minolta, Tokyo, Japan) with a D65 illuminant and CIE L* a* b* uniform color space, where L* represents lightness and darkness, a* indicates chromaticity on a green (−a*) to red (+a*) axis, and b* indicates the chromaticity on a blue (−b*) to yellow (+b*) axis. The analyses were performed in triplicate, with five measurements in each sample unit. Extracts were evaluated throughout in terms of redness index (a*/b*), Chroma (C* = (a*^2^ + b*^2^)^0.5^), and hue angle (*h*° = (arctan b*/a*)). The ratio a*/b* was considered a good indicator of color loss in beetroot puree. The hue expresses the color nuance and the values are defined as follows: red–purple = 0°, yellow = 90°, bluish-green = 180°, and blue = 270°. The C* is a measure of chromaticity, which denotes the purity or saturation of the color; the higher the chroma values, the higher the perceived color intensity of the samples to the naked eye [26].

### 2.7. Statistical Analysis

Data were analyzed for statistical significance using a two-way analysis of variance (ANOVA) and a one-way ANOVA to check for an effect of the single factors (total dose of enzymatic mix and processing time) or an interaction with extraction yield and color parameters (*p* < 0.01). A Tukey’s honestly significant difference (HSD) post-hoc test (*p* < 0.05) was carried out by the EXCEL^®^ Add-in macro DSAASTAT program [27]. A Pearson correlation analysis was carried out on extraction yield and color attributes (expressed as “total betalain content” (Bt + Bx)); it was computed with GraphPad Prism 5.0 (GraphPad software, Inc., La Jolla, CA, USA) at a minimum significance level of *p* < 0.05.

## 3. Results and Discussion

### 3.1. Chemico-Physical Properties of Red Beets and Optimization of the Extraction Conditions

Unsold red beets, used in this study for the recovery of betalains, were first analyzed in terms of moisture, DM, pH, TA, total sugars, and cell-wall polysaccharide composition. The main chemico-physical parameters (in wet weight basis (_wwb_)) of red beets were consistent with those reported in the literature [21] and were as below: moisture 89 ± 1%_wwb_; DM 11 ± 1%_wwb_; pH 6.11 ± 0.01; TA 0.14 ± 0.03 (gcitric acid/100 g_wwb_); and total sugar content of 12 g/100 g_wwb_. These data demonstrate that the red beet used had the optimal ripening stage, which corresponds to the highest betalain amount [28].

The cell-wall polysaccharide composition (in dry weight basis (_dwb_)) was: cellulose 37% (4.0 ± 0.1 g/100g_dwb_), pectin 28% (3.1 ± 0.1 g/100g_dwb_), and hemicellulose 35% (3.7 ± 0.1 g/100g_dwb_). Cellulose was the predominant component, followed by hemicellulose and pectin. Zieminski et al. [29] and Spagnuolo et al. [30] reported similar results, highlighting that red beet pulp may contain 22%–30% cellulose and 24%–32% pectin, depending on variety. Hence, bearing in mind the red beet cell-wall composition, a tailored enzymatic mix (CL: 37%; PG and PL: 28%; and XL: 35%) was fine-tuned. The proportion of each enzyme preparation in the multi-component mix was calculated with consideration to the corresponding enzymatic units (I.U./g).

**Figure 1:**
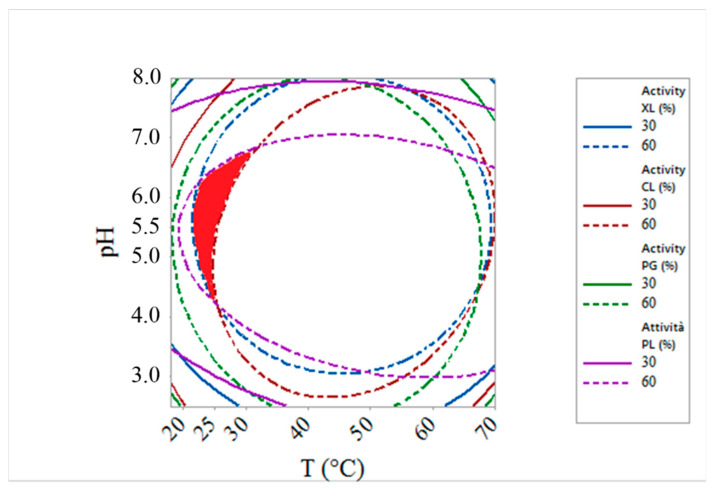
The optimum region, determined by overlaying contour plots of relative enzyme activity (polygalacturonase and pectin-liasic (PG + PL), cellulase (CL), and xylanase (XL)), evaluated as a function of temperature and pH. The region highlighted in red is the area in which enzyme preparations PG + PL and XL preserved a relative activity > 60%, whereas CL showed a relative activity between 30 and 60% (temperature 20–25 °C and pH 4.5–6.5) (from Lombardelli et al., 2020 [5]).

The optimal conditions (temperature (T) and pH) for maximizing the activity of all the enzymes forming the tailored mix, assessed by response surface methodology analysis, were: temperature between 45 and 60 °C and pH between 5 and 6, as demonstrated in a previous study [5]. Thus, all the extraction experiments were carried out at 45 °C and pH 5.5. It is known that, under those conditions, betalains are quite stable [14]. Moreover, taking into account the strong influence of temperature on betalain stability (in particular for betacyanin), other experiments were conducted at 25 °C, considering that between 20 and 25 °C (pH 4.5–6.5) enzyme preparations PG + PL and XL preserved a relative activity greater than 60% and CL showed a relative activity between 30 and 60%, as shown by the overlay contour plot (Figure 1).

### 3.2. Influence of Different Enzyme Mix Dosages, Processing Times, and Temperatures on Betalain Extraction 

Nowadays, the recovery of stable natural pigments from food-waste-based matrixes is gaining attention, as described by Galanakis [31], Gupta et al. [32], and Ngamwonglumlert et al. [33]. This research suggests a need for a tailored protocol based on a targeted substrate-enzymatic extraction that avoids the use of organic solvents for the recovery of betalains from unsold red beets. The specific enzymatic mix was designed on the basis of red beet cell-wall composition (CL: 37%, PG and PL: 28%, and XL: 35%), and was applied at 45 and 25 °C, at pH 5.5. The processing time (20−300 min) and total dose of enzymatic mix (10–50 U/g) were fine-tuned to improve the betalain recovery yield (in terms of Bx and Bt).

**Figure 2:**
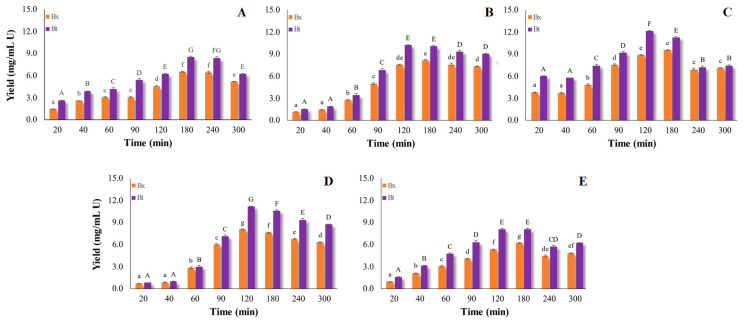
Extraction yield of betalains from unsold red beets at 45 °C and pH 5.5. Trials were performed using different total doses of enzymatic mix: (**A**) 10 U/g, (**B**) 18 U/g, (**C**) 25 U/G, (**D**) 38 U/g, and (**E**) 50 U/g for several processing times. For betacyanins (Bt), means, indicated by capital letters, vary significantly (one-way variance analysis, *p* = 0.05) with processing time. For betaxanthins (Bx), means, indicated by lower-case letters, vary significantly (one-way variance analysis, *p* = 0.05) with processing time.

**Figure 3:**
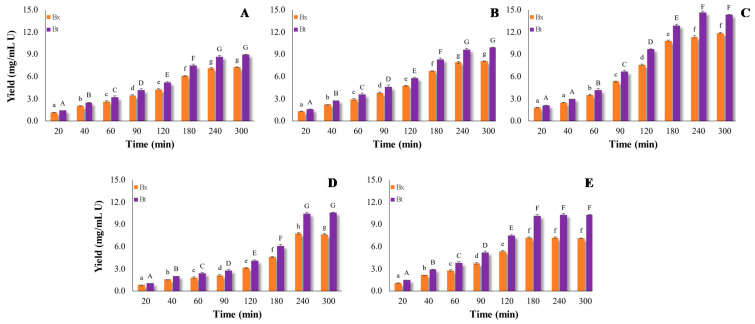
Extraction yield of betalains from unsold beetroot at 25 °C and pH 5.5. Trials were performed using different total dosages of enzymatic mix: (**A**) 10 U/g, (**B**) 18 U/g, (**C**) 25 U/G, (**D**) 38 U/g, and (**E**) 50 U/g, at several processing times. For betacyanins (Bt), means, indicated by capital letters, vary significantly (one-way variance analysis, *p* = 0.05) with processing time. For betaxanthins (Bx), means, indicated by lower-case letters, vary significantly (one-way variance analysis, *p* = 0.05) with processing time.

The two-way ANOVA (total dosage vs. processing time) demonstrated that Bx and Bt recovery yields were significantly affected by the investigated factors (*p* < 0.01, data not shown). Moreover, data analyzed by means of one-way ANOVA (Figure 2 and Figure 3) enabled us to test for significant differences between treatments involving each factor (total dosage or processing time). For all the tested total dosages (10–50 U/g), and regardless of the temperature applied (45 or 25 °C), the Bx and Bt yield gradually increased at longer processing times, until reaching the highest value after 120 min at 45 °C (Bx = 8.89 ± 0.03 and Bt = 12.15 ± 0.03 (mg/L)/U) (Figure 2) and after 240 min at 25 °C for the dosage 25 U/g (Bx = 11.37 ± 0.45 and Bt = 14.67 ± 0.49 (mg/L)/U) (Figure 3). These data prove that the enzymatic hydrolysis of cell-wall components was faster at 45 °C compared with 25 °C, and mainly occurred within the first 2 h of incubation in all samples. The only exception appeared to be the treatment 10 U/g (Figure 2A), which required about 3 h to achieve the highest betalain extraction yield.

After 2 h at 45 °C, the recovery yield decreased, probably due to the weak stability of Bt, which is strongly affected by temperature (Figure 2). In this regard, Havlikova et al. [34] proved that during the heating (50 °C) of betanin (a component of Bt), the molecule is split, giving rise to cyclodopa and betalamic acid. Meanwhile, data in Figure 3 prove that at 25 °C and with longer processing times, the extraction yield did not decrease and no differences among samples were found. López et al. [35] reported that the extraction of betalains by pulsed electric fields at 30 °C was more efficient than at 60 °C, which can be explained by temperature-dependent pigment degradation reactions. Moreover, the betacyanin/betaxanthin ratio was not stable at 45 °C during longer treatment times, while no changes in this ratio were revealed at 25 °C (Figure 2 and Figure 3). Prieto-Santiago et al. [26] explained that the variation in this ratio could be ascribable to the degradation of Bt, as well as to the formation of Bx. Indeed, the latter could arise from the condensation of free amino acids with the betalamic acid, generated by Bt hydrolysis [26]. 

Regardless of the extraction temperature (45 or 25 °C), it is suggested that, at the lower tested dosages (10 and 18 U/g), the small amount of pectinase activity may be not adequate to break down the pectin network and release pigments. Both at 45 °C and at 25 °C, the minimal suitable total dosage was 25 U/g. More specifically, at 45 °C, the extraction yield ranged from 3.83 ± 0.01 to 8.89 ± 0.03 after 20 and 120 min for Bx, and from 6.03 ± 0.01 to 12.15 ± 0.03 after 20 and 120 min for Bt (Figure 2). Furthermore, at 25 °C, an extraction yield ranging from 1.82 ± 0.01 to 11.37 ± 0.45 after 20 and 240 min for Bx was observed, and from 2.14 ± 0.01 to 14.67 ± 0.67 for Bt after 20 and 240 min (Figure 3).

When a higher enzyme-mix dosage (38 and 50 U/g) was used, the extraction yields decreased with respect to 25 U/g, probably due to the fast hydrolysis degree, which may have led to inhibition of the end-product [36,37].

Overall, the optimal extraction conditions for maximizing the recovery yield of Bt and Bx appeared to be 25 °C for 240 min with an enzymatic-mix dosage of 25 U/g.

### 3.3. Color Characterization of Betalain Extracts

**Figure 4:**
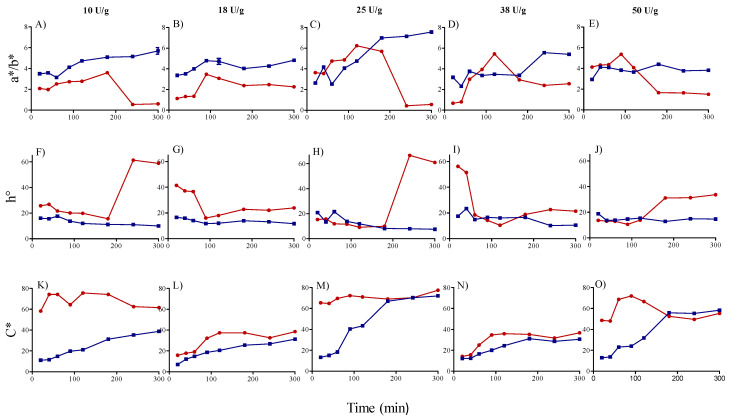
Color parameters (**A**–**E**), a*/b* (**F**–**J**), hue angle (*h*°) (**K**–**O**), Chroma value (C*) of betalain extract recovered at 45 °C (●) and 25 °C (■), using different total doses of enzymatic mix (10–50 U/g).

The preservation of color properties is crucial in the development of a novel extraction protocol for the recovery of colorants from plant-based matrices. The color parameter’s red–yellow ratio (a*/b*, indicating the redness of the extract), chroma (C*, showing the dullness/vividness of the product), and hue angle (*h°*, the color perception by human eye), considered to be the best descriptors of color, were used to characterize the different betalain extracts recovered by the tailored enzymatic mix (Figure 4).

Their values were in agreement with those reported in the literature (Prieto-Santiago et al., 2020) [26]. At 45 °C, regardless of the dosage used, a*/b* increased as the extraction time increased until reaching the maximum value, beyond which a strong decrease at longer extraction times (in particular at 240 and 300 min) was revealed. A similar trend was observed at 25 °C, despite a*/b* not decreasing at longer extraction times (Figure 4A–E). In particular, the highest values for the betalain extracts were recorded using the dosage 25 U/g after 120 min at 45 °C (6.24 ± 0.04) and after 240 min at 25 °C (7.15 ± 0.01) (Figure 4A–E).

Observed *h*° angle values were always lower for the extracts recovered at 25 °C than for those obtained at 45 °C. More specifically, at 45 °C, the hue decreased slightly as the extraction time increased, and then significantly increased with longer times (240 and 300 min) irrespective of the dosage used. At 25 °C, this parameter significantly decreased as the extraction time increased, and then remained constant (Figure 4F–J). Considering that red–purple color has an *h*° near 0°, the best values of *h*° were revealed for the betalain extracts recovered using the dosage 25 U/g after 120 min at 45 °C (9.11 ± 0.05) and after 240 min at 25 °C (7.96 ± 0.01) (Figure 4F–J). 

The behavior of the above-described color parameters (a*/b* and *h*°) could be explained by the aforementioned betacyanin degradation reactions, which resulted in a differential extraction of betacyanin vs. betaxanthin fractions (Figure 3). The thermal hydrolysis reactions at 45 °C not only produced a decrease in tinctorial strength, but also a considerable color shift towards yellow nuance [26].

Concerning C*, no clear trend was observed at 45 °C, whereas at 25 °C (regardless of the dosage used), a gradual rise in this parameter was evident as extraction time increased. The extracts recovered using the dosage 25 U/g were characterized by similar values of C*, despite the processing time at 25 °C (C* = 70.30 ± 0.08 after 240 min) being 2 times longer than that at 45 °C (C* = 71.02 ± 0.78 after 120 min).

In order to suggest a novel, green, and sustainable biotechnological approach for the recovery of betalains from unsold red beets, the effects of enzymatic-mix dosage, processing time, and temperature on extraction yield and color attributes were considered. Accordingly, a correlational analysis between both dependent variables (extraction yield and color attributes) was conducted. Taking into account that measured color is determined by the contribution of both betacyanins and betaxanthins, the variable “total betalain content” (Bt+Bx) was used in the statistical analysis.

The color parameters a*/b*, chroma, and hue angle always appeared correlated (negatively or positively) with the total betalain content, regardless of the temperature and mix total dosage used (Table 1). Color parameters showed high correlation coefficients with the total betalain content at 25 °C compared with 45 °C, and especially when a total dosage of 25 U/g was applied. In this case, a*/b* and C* showed the strongest positive correlations (0.9478 and 0.9802), whereas *h*° angle had the strongest negative correlation (−0.8823) (Table 1). Accordingly, our results indicate that the a*/b* ratio, hue angle, and chroma values may be linked to a higher betalain extraction yield.

Overall, the data reported in this paper, as well as the strong Pearson correlation coefficients, prove that the most suitable extraction conditions (in terms of yield and color attributes) for the recovery of betalains from unsold red beets are: a 25 U/g total dose of enzymatic mix; a temperature of 25 °C, and a processing time of 240 min.

## 4. Conclusions

The novel and significant contribution of the current research is the demonstration that the recovery of betalain from unsold red beets can be improved by applying a specific enzymatic mix based on the polysaccharide composition of the red-beet cell wall. Although the pigment extraction at higher temperatures (45 °C) was faster, a comparable recovery yield of betacyanin and betaxanthin fractions was achieved at lower temperatures (25 °C). Moreover, the low-temperature enzyme-assisted extraction protocol allowed better preservation of the color attributes of the betalain extracts.

Overall, the optimal conditions for the extraction of betalain from unsold red beets were: 25 U/g total dose of enzymatic mix, a temperature of 25 °C, and a processing time of 240 min. These results prove that betalain recovery could be a “green route” to coloring foodstuffs, giving much greater value to unsold red beets.

## Figures and Tables

**Table 1:** Pearson correlation coefficients (*p* < 0.05) between total betalain content and color parameters in beetroot extracts for different enzyme-mix total dosages (10–50 U/g) at different temperatures (45 and 25 °C), estimated for the whole processing time (0–300 min).

Dosage (U/g)	Color Parameter	Betalain Content (45 °C)	Betalain Content (25 °C)
10	a*/b*	−0.0968	0.8077
	*h*°	0.3760	−0.8050
	C*	−0.0235	0.9505
18	a*/b*	0.7539	0.6181
	*h*°	−0.8694	−0.6557
	C*	0.9364	0.9612
25	a*/b*	0.6607	0.9478
	*h*°	−0.1613	−0.8823
	C*	0.6392	0.9802
38	a*/b*	0.7576	0.8928
	*h*°	−0.8286	−0.8043
	C*	0.873	0.9112
50	a*/b*	−0.4401	0.4122
	*h*°	0.4852	−0.4480
	C*	−0.0059	0.9026

## Data Availability

The data presented in this study are available on request from the corresponding author.
